# Effect and cost of an after-school dance programme on the physical activity of 11–12 year old girls: The Bristol Girls Dance Project, a school-based cluster randomised controlled trial

**DOI:** 10.1186/s12966-015-0289-y

**Published:** 2015-10-06

**Authors:** Russell Jago, Mark J. Edwards, Simon J. Sebire, Keeley Tomkinson, Emma L. Bird, Kathryn Banfield, Thomas May, Joanna M. Kesten, Ashley R. Cooper, Jane E. Powell, Peter S. Blair

**Affiliations:** Centre for Exercise, Nutrition and Health Sciences, School for Policy Studies, University of Bristol, 8 Priory Road, Bristol, BS8 1TZ UK; Bristol Randomised Trials Collaboration, School of Social and Community Medicine, University of Bristol, Bristol, BS8 2PS UK; Health and Social Sciences, University of the West of England, Bristol, UK

**Keywords:** Physical activity, Dance, School, RCT

## Abstract

**Background:**

The aim of this study was to examine the effectiveness and cost of an after-school dance intervention at increasing the physical activity levels of Year 7 girls (age 11–12).

**Methods:**

A cluster randomised controlled trial was conducted in 18 secondary schools. Participants were Year 7 girls attending a study school. The Bristol Girls Dance Project (BGDP) intervention consisted of up to forty, 75-minute dance sessions delivered in the period immediately after school by experienced dance instructors over 20-weeks. The pre-specified primary outcome was accelerometer assessed mean minutes of weekday moderate to vigorous physical activity (MVPA) at time 2 (52 weeks are T0 baseline assessments). Secondary outcomes included accelerometer assessed mean minutes of weekday MVPA at time 1 (while the intervention was still running) and psychosocial outcomes. Intervention costs were assessed.

**Results:**

571 girls participated. Valid accelerometer data were collected from 549 girls at baseline with 508 girls providing valid accelerometer data at baseline and time 2. There were no differences between the intervention and control group for accelerometer assessed physical activity at either time 1 or time 2. Only one third of the girls in the intervention arm met the pre-set adherence criteria of attending two thirds of the dance sessions that were available to them. Instrumental variable regression analyses using complier average causal effects provided no evidence of a difference between girls who attended the sessions and the control group. The average cost of the intervention was £73 per girl, which was reduced to £63 when dance instructor travel expenses were excluded.

**Conclusion:**

This trial showed no evidence that an after-school dance programme can increase the physical activity of Year 7 girls. The trial highlighted the difficulty encountered in maintaining attendance in physical activity programmes delivered in secondary schools. There is a need to find new ways to help adolescent girls to be physically active via identifying ways to support and encourage sustained engagement in physical activity over the life course.

**Trial registration:**

ISRCTN52882523

**Electronic supplementary material:**

The online version of this article (doi:10.1186/s12966-015-0289-y) contains supplementary material, which is available to authorized users.

## Introduction

Among adults, physical activity is associated with reduced risk of heart disease, type 2 diabetes and improved psychological well-being [1]. Among children and adolescents physical activity is associated with a lower prevalence of obesity, lower blood pressure, lower lipid profile and improved psychological well-being [2]. Physical inactivity, defined as less than 600 MET minutes per week, is also the tenth main cause of disability adjusted life years globally [3]. Several recent articles have focussed on the dose response relationship between physical activity and health and suggest that relatively small increases in physical activity at a population level would result in marked reductions in all-cause mortality [4, 5]. Data from the UK Millennium cohort study has shown that only 51 % of seven year olds met the recommendation of an hour of moderate-to-vigorous intensity physical activity (MVPA) per day [6]. The amount of physical activity in which children engage reduces as they progress through primary and secondary school, with girls being less active than boys [7, 8]. These patterns also have the potential to track from childhood to adulthood [9]. The start of secondary school (11–12 years of age) is a critical period of change in the physical activity levels of girls [7]. As such, finding ways to help girls to be more active at the start of secondary school is important for short and long-term health.

Schools provide opportunities to implement public health interventions to large numbers of adolescents [10, 11]. A number of interventions have attempted to increase adolescent physical activity at school, however, systematically reviewed evidence has indicated that the effectiveness of school-based physical activity interventions delivered during the curriculum is limited [12]. The review concluded that where there was evidence of an effect, it was mainly due to the results of studies with poor methodological quality [12]. Identified limitations included short duration of follow-up, inadequate adjustment for potential confounders, lack of adjustment for children clustered in schools, and the use of self-report measures of physical activity [12]. A 2012 review of physical activity interventions for children and adolescents, which included an objective assessment of physical activity reported an average improvement of four minutes of MVPA per day in intervention participants when compared to control groups [13]. Of the 30 studies included in the review, only 16 were deemed to be of high methodological quality. Contributory factors to low quality scores included high attrition, lack of intention to treat analyses, and not adjusting for the clustered nature of the data. A 2015 meta-analysis of physical activity interventions with adolescent girls showed that public health focussed interventions can be effective but the intervention effect was enhanced if the interventions included only girls, was school-based and employed a theory of behaviour change [14]. Thus, tailoring interventions to the interests and needs of girls is likely to enhance effectiveness.

Extra-curricular interventions can exploit the space, facilities and infrastructure of schools to provide opportunities for children to be physically active [10, 15, 16]. A 2009 narrative systematic review identified 11 studies that had attempted to increase physical activity via extra-curricular programmes [15], only six studies reported effects on physical activity [15]. The review also identified a lack of information about programme adherence, intervention components and the effect of attending after-school programmes on overall levels of physical activity. Only six out of nine studies (all conducted in North America and Australasia), identified by another review, provided any data from a period that was at least 12 weeks after the baseline assessment [17]. The authors concluded that lack of statistical power hindered the ability of the studies to assess the intervention effectiveness. After-school is a key period for extra-curricular interventions but these interventions differ to lunch-time programmes as they require participants to remain at school rather than being a captive audience. In preparing this paper, we conducted a literature search of randomised controlled trials involving after-school interventions aimed at increasing child and adolescent physical activity. Our review identified only four further randomised controlled trials [18–21] that have been published since 2011 [17] and two of these were feasibility trials conducted by our team [20, 21]. Only our own feasibility studies [20, 21] provided follow-up measurements to determine the impact on physical activity levels after the intervention had ceased, with only the dance feasibility showing sustained evidence of promise for an intervention effect [20].

Dance is the preferred form of physical activity for many UK secondary school aged girls [22]. Dance is a social activity, combining movement with group activities and music, and provides unique opportunities to help girls to be active. Recent studies have suggested that dance can positively effect a range of psychosocial factors [23–25], health outcomes [23] and contribute significantly to the overall MVPA of girls [26]. Additionally, dance is seen as a desirable and fun activity for adolescent girls [22, 27–29]. We previously reported the results of a feasibility trial in which we showed that an after-school dance programme can have the potential to increase the physical activity levels of secondary school aged girls [20].

Complex behavioural interventions based on theories of behaviour change have had more success than non-theory based interventions [30]. It has been proposed that intervention effectiveness is enhanced by targeting the key correlates of behaviour and then manipulating them within the intervention [31]. Self-determination theory (SDT)[32] may help to explain physical activity participation. Research using SDT among children shows that physical activity motivation that is autonomous (based on enjoyment or valued benefits) versus controlled (based on guilt or compliance with external demands) is associated with their physical activity, and that autonomous motivation is underpinned by the satisfaction of psychological needs for autonomy, competence and relatedness [33]. Dance is an activity which could progressively increase girls’ perceived autonomy to be active (they can be active when and where they want as minimal equipment is needed), increase their competence (skills can be built quickly), and foster meaningful connections with others in a social environment.

It is important to recognise that commissioners have limited resources to improve health of children in schools and as such they need to know whether investments in after-school physical activity programmes are affordable within a set budget [34]. Thus, there is a need for robust evaluations of the effectiveness and cost-effectiveness of paediatric physical activity interventions.

In this paper, we report the results of the Bristol Girls Dance Project (BGDP). The intervention aimed to increase the time spent in weekday MVPA among Year 7 girls via an after-school dance intervention. The BGDP trial used a study design that addressed many of the limitations of previous studies in this area. Specifically, schools were randomly allocated to intervention or control arm after baseline data had been collected, there was an objective assessment of physical activity using accelerometers, study staff collecting and analysing data were blinded to allocation, the primary analysis was based on intention to treat with the models adjusted for the clustering of children within schools.

## Methods

### Study design

BGDP was a school-based cluster randomised controlled trial. The trial protocol was published in 2013 prior to participant recruitment and data collection [35]. A more detailed trial analysis plan was developed and approved at our Trial Steering Committee meeting on 4/7/2014 (before the analysis team had access to any data). All analyses have followed the agreed analysis plan. The trial was registered at the controlled trial register prior to data collection (ISRCTN52882523).

### Eligibility and Recruitment

Participants were Year 7 (age 11–12) girls, hitherto referred to as ‘girls’ throughout. All mainstream state secondary schools in the constituent Local Authorities (LA) (Bath and North East Somerset, Bristol City, and North Somerset) were invited to participate in the study. Postal and email invites were sent to relevant staff in all schools and follow-up phone calls were made. Schools were excluded at the outset if they had less than 30 Year 7 girls or if they were a specialist Dance Academy.

As part of the participant recruitment process all Year 7 girls in 22 schools were provided with “taster sessions” of dance content during their regular physical education classes. These sessions were designed to engage low active girls by demonstrating that dance was a fun and social activity that the girls could engage in regardless of skill or previous dance experience. All taster sessions followed a standard structure and were delivered by independent dance instructors who were employed by the study. A total of 65 taster sessions were delivered in 22 schools that were recruited (One reserve school did not receive any taster sessions). At the end of the taster session girls received a briefing on the aims of the study and its design, along with parent and participant information sheets. If fewer than 25 children enrolled in a school (after multiple recruitment attempts) the school was withdrawn and replaced with a reserve school (*n* = 4). When more than 33 children signed up to the study in a given school, children were randomly ranked and the first 33 were selected to participate by computer algorithm. Children who dropped out before baseline data collection were replaced by reserves when possible. No replacements were made after baseline measurements.

Ethical approval was obtained from the School for Policy Studies ethics and research committee at the University of Bristol (ref: Bristol Girls Dance Project). Written parent consent was obtained for all children who wished to participate in the study. Children received a £10 “Love to Shop” voucher as a reimbursement for their time at each of the three data collections.

### Randomisation

Randomisation occurred at the school-level. Schools were randomly allocated in a ratio of 1:1 to intervention or control group with nine schools in each trial arm. Balance between trial arms was achieved according to four variables: Local Authority membership, average baseline MVPA, school size, and deprivation (measured as the percentage of pupils in schools eligible for the Department of Education’s Pupil Premium [36]).

### Intervention

The aim of BGDP intervention was to increase MVPA among Year 7 girls by increasing their exposure to dance via an after-school intervention. We hypothesised that attending the programme would increase girls’ autonomous motivation through increased perceptions of autonomy, competence to be active and belonging to an active group of peers. It was hypothesised that dance programme attendance would provide increases in habitual physical activity while the programme was running. Both improvements were hypothesised to further support intervention girls’ sense of autonomy, competence and belongingness towards being active, which would facilitate their continued activity once the intervention ended. Girls in the control schools provided data only. Control schools received a £500 donation once all data had been collected.

#### What the intervention involved, including who delivered the different aspects of it

Full details of the intervention components have been reported elsewhere [35] and are summarised in Additional file 1: Table A which reports intervention components in accordance with the TIDieR guidelines [37]. Briefly, the intervention consisted of up to forty, 75-minute dance sessions provided twice per week between January and July 2014. Session plans included guidance on how to reinforce the underpinning SDT principles, and advice on activities, group work and dance skill development. To reflect a ‘normal’ dance session, instructors were able to decide on the genre of dance used, after consultation with the girls in their school. The nine intervention schools were asked to complete as many of the 40 sessions as possible before the end of the school year. Dance sessions were delivered at the school site in appropriate facilities. All sessions were delivered by experienced dance instructors who had undergone a one day induction session. Around the mid-point of the intervention period (April) dance instructors attended a half day booster session which recapped the study objectives and reinforced the motivational principles of SDT.

Ten instructors delivered the intervention. Instructor absences were covered by reserve instructors/those delivering the intervention in different schools. Due to work commitments one instructor withdrew from the study midway through the intervention period and was replaced by a reserve. One instructor delivered the intervention in two schools.

### Participant assessments

Baseline (T0) assessments were undertaken between September and November 2013 (prior to randomisation) when girls were in the first term of Year 7. The first follow-up (T1) was conducted during weeks 17–20 of the intervention and was designed to provide an assessment of MVPA during the intervention. The second follow-up (T2) was undertaken approximately 52-weeks after T0 assessments (all T2 assessments were undertaken within 3 weeks of the 52-week target and were all at least 4 months after the intervention had ended). Trained fieldworkers who were blinded to school intervention allocation collected all data.

The primary and secondary outcome measures assessed at all three time points are listed in Table 1. Physical activity was assessed using an Actigraph GT3X+ accelerometer. Participating girls wore an accelerometer for seven days. Accelerometer data were processed to identify days in which valid data were provided. Based on established protocols a valid day of accelerometer data was defined as a minimum of 500 minutes of data between 05:00 and 11.59 pm. Periods of 60 minutes or more in which zero values were recorded were interpreted as ‘non-wear’ time. For valid days, the mean minutes engaged in MVPA (≥2296 counts per minute) [38] and the mean accelerometer counts per minute (an indication of the average intensity in which girls engaged) were derived. The following accelerometer variables were then obtained: weekday and weekend day counts per minute, mean weekday and weekend day minutes of MVPA, and mean weekday sedentary time. Girls were included in the analysis if they provided two valid weekdays of data or one valid weekend day for the weekend variables.

**Table 1:** Primary and secondary outcomes

Primary outcome	Accelerometer-assessed mean weekday minutes of MVPA per day 12-months after baseline assessment (T2)
Secondary outcomes	Accelerometer-assessed mean weekday minutes of MVPA per day during the intervention period (T1)^a^
	Mean weekend minutes of MVPA at T1 & T2
	Mean weekday accelerometer counts per minute at T1 & T2
	Mean weekend accelerometer counts per minute at T1 & T2
	Proportion of girls meeting recommended 60 minutes of MVPA per day at T1 & T2
	Mean accelerometer-derived minutes of weekday sedentary time at T1 & T2
	Costs of delivering the intervention
	Physical activity motivation and psychological need satisfaction at T1 and T2
	Health related quality of life (EQ-5DY)

^a^Key secondary outcome

*MVPA* Moderate-to-vigorous physical activity

*EQ*-*5D*-*Y* European Quality of Life-5 Dimensions (Youth version)

T1 – 20-week follow-up

T2 – one-year follow-up

Girls completed a 67-item psychosocial questionnaire which assessed autonomous and controlled motivation for dance and PA [39], perceptions of autonomy, competence and relatedness [40, 41] within PA, and self-esteem [42]. Girls also completed an EQ-5D-Y form at each time point [43] as a measure of health-related quality of life.

For descriptive purposes, at T0 parents/guardians reported on their own ethnicity, highest level of household education and home address. The index of multiple deprivation (IMD) was calculated based on each girl’s home postcode. Height was assessed to the nearest 0.1 cm and weight to the nearest 0.1 kg using a Seca stadiometer and Seca digital scale, respectively. Participant body mass index (BMI = kg/m^2^) was calculated and converted to an age and gender-specific standard deviation score [44]. The after-school activities in which girls engaged were obtained via parent report at T0 with after-school and weekend participation in activities self-reported by the girls at T1 and T2. Attendance was recorded by dance instructors at each session. The results of an in-depth process evaluation of the study will be reported separately and a link to the study paper placed on the project website (http://www.bristol.ac.uk/sps/research/researchprojectpages/active7/) when available. Briefly however, the mixed-methods evaluation indicated that the girls enjoyed the dance sessions and the decrease in attendance was largely attributed to factors outside of the dance sessions. Fidelity to the underlying theoretical principles was moderate; girls felt that the instructors provided good support for their competence and relatedness and that there was room for improvement in the extent to which instructors supported the girls’ autonomy. Dance instructors, school contacts and the girls also indicated that two sessions per week was perhaps too large a commitment.

Reporting of resource use and cost estimation are in accordance with relevant categories of the CHEERS checklist [45]. Data on resource use were collected by the project team and recorded using an existing checklist [46]. Costs were categorised as one-off training costs, recurrent programme preparation costs, recurrent programme delivery costs, and were stratified by school. Recruitment and marketing costs were identified separately because they depend upon the implementation context for participation in each school setting [47]. These costs might have differential timing at initiation of mainstream delivery and/or may not always apply in practice [45]. Prices were taken from actual costs on time sheets, published and established sources.

### Sample size calculation

Sample size calculations were based on detecting a ten-minute difference per day in the habitual MVPA of the intervention group when compared to the control group. This difference was selected because a 2012 meta-analysis showed that a ten-minute change in MVPA would have significant impacts on children’s cardio-metabolic risk profile [48] and our feasibility trial showed that such an effect was achievable based on the 95 % confidence intervals [20]. The feasibility trial also suggested that a ten-minute MVPA change would increase the proportion of girls meeting current recommendation of 60 minutes of MVPA per day [1] from 8 to 17 %. The sample size calculations were inflated to take account of the clustering of girls in schools. In the feasibility trial the upper limit of the school associated intra-cluster correlation was 0.087. Thus, using an ICC of 0.087 and a final cluster size for analysis of 24 (20 % drop-out from a target of 30 girls per school) we estimated that with 90 % power and 5 % (two-sided) alpha an initial sample of 540 girls from 18 schools (30 per school) was required. To account for potential drop-out between data collection (autumn term 2013) and the intervention start (January 2014) we increased the maximum number of girls per school to 33.

### Statistical analyses

The statistical analysis plan was agreed by the project Trial Steering Committee prior to analyses being conducted. Descriptive statistics (mean, standard deviation, median, inter-quartile range, and percent) were used to describe the T0 data and levels of data provision at T1 and T2. Multi-variable mixed effects linear models were used to assess primary and secondary outcomes at T1 and T2. The primary analysis included weekday MVPA at T2 and included trial arm and weekday MVPA at T0. Variables used in the randomisation process (local authority, school size and school level percentage of deprivation) were also included in the model. This process was repeated for all secondary outcomes. A comparable logistic regression model was used to assess whether there was a difference in the proportion of girls who met the 60 minutes of MVPA per day guidance at T1 and T2. Models for weekday MVPA at T2 and T1 were re-run using a complier average causal effect (CACE) instrumental variable regression models [49]. The CACE models included all girls and used random allocation as an instrumental variable to calculate the effect of the intervention for those who adhered to it, by comparing those girls observed to attend the dance sessions with those in the comparison group who would have attended if invited [49]. Girls were considered to have adhered to the study protocol if they attended 2/3 of the sessions provided at their school. The CACE models were run once the analysis team had become un-blinded (2nd Feb 2015). All models were adjusted for the clustering of girls in schools and were conducted in Stata (version 13.1, College Station, TX).

Once the primary, secondary and CACE analyses had been completed, further exploratory analyses were undertaken to gain a fuller understanding of study findings. A rank-sum test was used to examine whether there was a difference in the individual level socio-economic position of girls between the intervention and control group. As there was some evidence (*p* < 0.01) of a difference in socio-economic position (intervention group having lower levels of deprivation), the primary and the key secondary outcome analyses (T1 weekday MVPA) were re-run with individual level IMD as a covariate. As there was minimal missing data and no evidence of a systematic bias in the proportion of missing data, we considered missing data to be missing completely at random and did not conduct further imputation models [50, 51].

To understand the amount of physical activity that was obtained during the dance sessions the accelerometer data were further examined to identify the mean minutes of sedentary, light, MVPA and mean CPM during the period that the dance sessions were scheduled to run at T1 (15:00 – 17:00). These data are included in Supplementary Table C for the girls who attended the dance sessions during the monitoring period on days of the dance classes (dance days) and for the day after the dance class (non-dance day). The same data were then presented for girls assigned to the intervention group who did not attend the dance classes and control group girls. Paired sample t-tests were used to examine differences between the accelerometer variables for dance days and non-dance days.

Based on UK Population Norms for EQ-5D, EQ-5D-Y responses from each time point were converted into utility scores ranging from 0.0 (dead) to 1.0 (perfect health) [52]. Mann Whitney U tests were used to examine differences between intervention and control group utility scores at each time point.

## Results

**Fig. 1:**
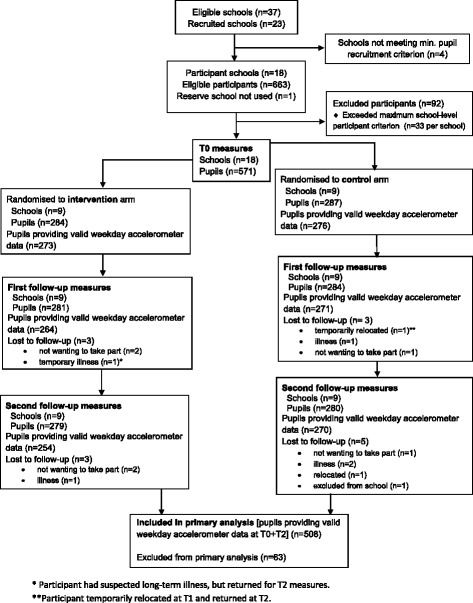
Trial profile for the Bristol girls dance project (CONSORT FLOW Diagram)

Figure 1 shows the trial profile. A total of 1877 Year 7 girls were eligible to participate in the 18 schools that formed the final study sample. 663 pupils from these schools provided consent to participate (35.3 % of the sample population), and 571 enrolled. Nine schools were over-subscribed (range = 34–62) and there was an average of 31.7 girls per school (range = 26–33). Data provision at each time point is shown in Additional file 1: Table B. 571 girls were randomized to the intervention or control arm with nine schools per trial arm. Of the 571 girls, there were 559 who provided some data at T2. 508 girls met the accelerometer inclusion criteria for the primary analysis. No adverse events were reported during the study.

Baseline (T0) characteristics (Table 2) show that almost 97 % of girls provided at least two days of valid weekday accelerometer data. Girls allocated to the intervention group performed an average of 53 minutes of weekday MVPA compared to 49 minutes in the control group.

**Table 2:** Baseline data descriptive statistics

	Control				Intervention			
Variable	*N*	Mean	SD or IQR		*N*	Mean	SD or IQR	
Mean height (cm)	287	149.44	7.42		284	149.27	7.04	
Median weight (kg)^c^	287	41.90	35.90 to 49.30		284	41.50	37.15 to 49.05	
BMI (kg/m^2^)	287	19.53	3.70		284	19.48	3.44	
BMI SDS^d^	285	0.38	1.21		279	0.40	1.16	
	N	Median	25 IQR	75 IQR	N	Median	25 IQR	75 IQR
IMD score summary statistic	287	17.99	9.81	35.35	282	15.68	9.29	23.91
Accelerometer provision	N^a^	N^b^	%		N^a^	N^b^	%	
Proportion with valid weekday data (≥2 valid weekdays)^e^	286	276	96.50		282	273	96.81	
Proportion with valid weekend day data (≥1 valid weekend days)	286	221	77.27		282	210	74.47	
Weekday accelerometer data	N	Median	25 IQR	75 IQR	N	Median	25 IQR	75 IQR
Total valid weekday mins	280	3519.17	2715.08	4027.58	280	3267.00	2571.50	3972.08
Average valid weekday mins	280	787.46	725.35	832.69	280	779.36	724.32	829.31
Total valid weekday CPM	280	431.78	352.53	523.02	280	476.30	396.17	555.07
Average weekday MVPA mins	280	49.15	37.38	60.65	280	53.25	41.50	68.03
Average weekday light PA mins	280	193.27	166.71	219.80	280	196.57	171.62	225.75
Average weekday sedentary mins	280	528.35	474.25	581.08	280	520.26	463.47	568.38
Weekend accelerometer data	N	Median	25 IQR	75 IQR	N	Median	25 IQR	75 IQR
Total valid weekend day mins	221	1238.50	655.00	1440.00	210	1215.33	660.50	1389.00
Average valid weekend day mins	221	694.75	618.17	759.17	210	675.96	609.75	736.08
Total valid weekend day CPM	221	378.64	296.24	533.03	210	416.18	324.03	558.45
Average weekend day MVPA mins	221	32.50	22.17	48.33	210	35.38	24.58	54.33
Average weekend day light PA mins	221	181.50	154.42	214.08	210	192.83	155.08	223.75
Average weekend day sedentary mins	221	476.58	398.00	529.42	210	437.25	384.83	492.50
Psychosocial variables	N	Mean	SD		N	Mean	SD	
Autonomous motivation dance	287	3.91	0.58		284	3.91	0.65	
Autonomous motivation PA	287	4.01	0.63		284	3.96	0.74	
Controlled motivation dance	287	1.95	0.72		284	1.94	0.63	
Controlled motivation PA	287	2.25	0.76		284	2.17	0.74	
Autonomy need satisfaction	287	5.67	1.00		284	5.62	1.05	
Competence need satisfaction	287	5.24	1.19		284	5.17	1.23	
Relatedness need satisfaction	287	5.89	1.26		284	5.89	1.31	
Self esteem	287	4.96	0.81		284	4.97	0.77	

N^a^ – N of participants with any valid accelerometer data

N^b^ – N of participants meeting inclusion criteria

^c^Median & IQR reported for weight as it is non normal

^d^Age adjusted BMI score. Missing data due to no date of birth being reported

^e^Percentage of girls who had ≥2 valid week days of accelerometer data at T0.

PA - Physical Activity

MVPA – Moderate-to-vigorous intensity physical activity

CPM - Counts per minute

The main intention-to-treat analyses with adjustment for baseline values found no difference in weekday MVPA at T2 in children allocated to the intervention group when compared to those allocated to the control group (Table 3). There was also no difference in the key secondary outcome of weekday MVPA at T1. There was no evidence of a difference in any accelerometer derived variables at T1 or T2 (Table 4). 81 girls with valid accelerometer data at T0 and T2 met the adherence criteria. Of the girls who provided valid accelerometer data at T0 and T1, 83 met the adherence criteria. The unadjusted mean (standard deviation) minutes of weekday MVPA at T2 was 53.6 (18.9) for the girls that adhered and 58.0 (23.1) for the girls that did not adhere. At T1 the means and standard deviations were 59.8 (21.6) for the adhered group and 60.8 (23.7) for the non-adhered group. The CACE per-protocol analysis found no evidence of a difference between the two groups (Table 3) for weekday MVPA at T2 or T1. Further sensitivity analysis (data not shown) yielded no evidence of changes to the findings after additional adjustment for individual level IMD.

**Table 3:** Means and standard deviations by trial arm and linear mixed model adjusted for imbalance at baseline for Weekday MVPA at T1 and T2

	Control			Intervention				
	*n*	Mean	SD	*n*	Mean	SD	Intervention vs Control adjusted difference in means (95 % CI)*	*P* value
T2 Mean weekday MVPA^a^	262	53.15	19.61	246	56.55	21.92	−1.52 [−4.76 to 1.73]	0.359
T1 Mean weekday MVPA^b^	265	57.69	19.39	256	60.46	22.98	−1.52 [−5.03 to 1.98]	0.395
T2 MVPA weekday CACE analysis	508	-	-	-	-	-	−4.79 [−14.53 to 4.96]	0.336
T1 MVPA weekday CACE analysis	521	-	-	-	-	-	−4.86 [−18.41 to 6.91]	0.365

^a^Primary comparison

^b^Key secondary outcome

*For between group differences the control group is the reference group with models adjusted for baseline mean weekday MVPA, Local Education Authority, school size, school level deprivation, school level baseline MVPA, the number of total valid week days at T0, the number of total valid week days at T2 (or T1) and school-level clustering

*MVPA* moderate-to-vigorous physical activity

*CACE* complier Average Casual Effect (Instrumental variable regression model).

T1 – 20-week follow-up

T2 – one-year follow-up

**Table 4:** Means and standard deviations by trial arm and linear mixed model adjusted for imbalance at baseline for accelerometer assessed secondary outcomes at T1 and T2

	Control			Intervention				
	*n*	Mean	SD	n	Mean	SD	Intervention vs Control adjusted difference in means (95 % CI)*	*P* value
	T2							
Mean weekend day minutes of MVPA	145	36.56	26.16	124	39.65	23.21	−1.75 [−7.51 to 4.01]	0.552
Mean weekday CPM	262	446.83	137.81	246	478.75	144.94	−2.44 [−25.25 to 20.38]	0.834
Mean weekend CPM	145	405.04	228.96	124	450.98	263.89	−4.11 [−61.07 to 52.86]	0.888
Proportion of girls meeting 60 mins MVPA per weekday ^a^	262	0.32	0.47	246	0.39	0.49	−1.18 [ −1.82 to 0.76]	0.458
Proportion of girls meeting 60 mins MVPA per weekend day ^a^	145	0.12	0.33	124	0.15	0.36	−1.11 [−2.39 to −0.52]	0.787
Mean weekday sedentary mins	262	533.01	80.54	246	515.12	80.22	−6.79 [−23.60 to 10.03]	0.429
Mean weekend sedentary mins	145	475.14	95.22	124	463.66	105.92	0.62 [−22.42 to 23.66]	0.958
	T1							
Mean weekend day minutes of MVPA	159	42.57	27.71	130	48.92	32.19	1.26 [−5.70 to 8.22]	0.723
Mean weekday CPM	265	500.35	177.32	256	529.42	157.72	−7.48 [−35.06 to 20.11]	0.595
Mean weekend CPM	159	492.21	371.47	130	543.67	284.39	6.27 [−72.10 to 84.65]	0.875
Proportion of girls meeting 60 mins MVPA per weekday ^a^	265	0.42	0.49	256	0.47	0.50	−1.11 [−1.68 to −0.73]	0.637
Proportion of girls meeting 60 mins MVPA per weekend day ^a^	159	0.17	0.38	130	0.27	0.45	−0.82 [−1.56 to −0.43]	0.543
Mean weekday sedentary mins	265	522.96	85.46	256	502.91	87.01	−7.72 [−27.32 to 11.87]	0.449
Mean weekend sedentary mins	159	464.13	92.28	130	452.46	98.21	- 8.94 [−31.91 to 14.04]	0.446

*For between group differences the control group is the reference group with models adjusted for baseline value, LEA, school size, school level deprivation and school-level clustering

^a^Odds ratio presented in Coefficient column

*MVPA* moderate-to-vigorous physical activity

*CPM* counts per Minute

There was evidence of small differences in all of the motivation scores at T2, except autonomy need satisfaction and self-esteem, with higher scores in the control group. There was a similar pattern at T1 where there was some evidence of a difference for all variables except controlled motivation for dance and autonomy need satisfaction (Table 5).

**Table 5:** Psychosocial Regression Results for T1 and T2

	Control			Intervention				
	*n*	Mean	SD	*n*	Mean	SD	Intervention vs Control adjusted difference in means (95 % CI)*	P value
	T2							
Autonomous motivation for dance (0–4 scale)	280	3.59	0.84	279	3.33	0.92	−0.27 [−0.40 to −0.13]	<0.001
Autonomous motivation PA (0–4 scale)	280	3.86	0.80	279	3.49	0.98	−0.34 [−0.48 to −0.21]	<0.001
Controlled motivation dance (0–4 scale)	280	1.76	0.75	279	1.65	0.65	−0.11 [−0.22 to −0.01]	0.045
Controlled motivation PA (0–4 scale)	280	2.20	0.84	279	1.88	0.73	−0.29 [−0.42 to −0.18]	<0.001
Autonomy need satisfaction (1–7 scale)	280	5.56	1.19	279	5.42	1.33	−0.12 [−0.32 to 0.07]	0.217
Competence need satisfaction (1–7 scale)	280	5.03	1.29	279	4.78	1.42	−0.22 [0.42 to −0.02]	0.027
Relatedness need satisfaction (1–7 scale)	280	5.92	1.41	279	5.53	1.62	−0.40 [−0.64 to −0.16]	0.001
Self-esteem (1–6 scale)	280	4.88	0.86	279	4.76	0.94	−0.12 [−0.26 to 0.10]	0.070
	T1							
Autonomous motivation for dance (0–4 scale)	284	3.74	0.68	281	3.51	0.82	−0.23 [−0.35 to −0.12]	<0.001
Autonomous motivation PA (0–4 scale)	284	3.91	0.73	281	3.65	0.90	−0.23 [−0.35 to −0.10]	<0.001
Controlled motivation dance (0–4 scale)	284	1.75	0.68	281	1.70	0.65	−0.06 [−0.16 to 0.04]	0.262
Controlled motivation PA (0–4 scale)	284	2.16	0.77	281	2.01	0.77	−0.12 [−0.23 to −0.01]	0.041
Autonomy need satisfaction (1–7 scale)	284	5.61	1.06	281	5.43	1.28	−0.15 [−0.33 to 0.02]	0.091
Competence need satisfaction (1–7 scale)	284	5.17	1.32	281	4.84	1.43	−0.29 [−0.47 to −0.10]	0.003
Relatedness need satisfaction (1–7 scale)	284	5.82	1.43	281	5.41	1.69	−0.42 [−0.66 to – 0.18]	0.001
Self-esteem (1–6 scale)	284	4.93	0.85	281	4.75	0.90	−0.19 [−0.32 to −0.06]	0.004

*For between group differences the control group is the reference group with models adjusted for baseline value, LEA, school size, school level deprivation and school-level clustering

PA physical activity

Girls who attended dance classes during the measurement period obtained 4.7 more minutes of MVPA, 14.2 more minutes of light intensity activity and 258 more accelerometer counts per minute between 15:00 and 17:00 on dance days versus non-dance days (Additional file 1: Table C). For intervention girls who did not attend dance sessions on the measurement days, there was no evidence of differences in the MVPA, light activity or CPM on the days that dance clubs were running compared with non-dance club days. The levels of MVPA, light and CPM for non-attendees were also comparable to the activity levels of control group girls on these days. Thus, for girls who attended on dance days, there were differences in MVPA, light and CPM, but the differences in MVPA were comparatively small.

Descriptive information on the number and proportion of children attending a variety of after-school activities at T0 is shown in Additional file 1: Table D. There were no apparent differences between the two trial arms for any of these variables. The number and proportion of intervention and control arm girls attending any non-school dance session and the number of sessions per week attended is shown in Additional file 1: Table E. The table shows some evidence of a difference in dance participation between the two study arms at T1 (36 % control, 31 % intervention) which was inverted at T2 (30 % control, 34 % intervention). There were no clear differences between trial arms in the number and proportion of girls attending sport clubs, activity clubs, playing on their own or engaging in sitting down activities at T1 and T2 (Additional file 1: Table F) .

A breakdown of the intervention cost is shown in Table 6. The BGDP cost £21,613; $35,878; €26,152 (in 2013–14 prices) across 9 schools, with an average cost per school of £2,401; $3,985; €2,905 and a variation in cost of £104; $173; €126. The average cost per girl was £73; $120; €87 with a range of £68-£77; $113-$128; €82-€93, due to differences in the total number of girls recruited to the study at study initiation. Sensitivity analyses demonstrated the average cost per girl was reduced to £63; $103; €75 when dance instructor travel expenses were excluded. There was no evidence of differences in EQ-5D-Y utility scores in participants allocated to the intervention group compared with those allocated to the control group (Additional file 1: Table G).

**Table 6:** BGDP resources and costs

Category and description of resources	Unit cost *£*	Number of units	Total cost *£*	Mean *(SD)* cost per school *£* (*n* = 9)
Recruitment and marketing costs^a^			£6,573	£730
One-off training resources				
Lead dance instructor delivery of dance instructor induction training			£297	£33
Dance instructor induction training	£32/hour	32 hours	£1,024	£114 (£64)
Travel expenses for induction training^b^			£40	£4 (£6)
Lead dance instructor delivery of dance instructor booster training			£180	£20
Dance instructor booster training	£32/hour	26 hours	£832	£92 (£43)
Travel expenses for booster training^b^			£43	£5 (£6)
Recurrent programme preparation resources				
Printing - training guide	£3.20/guide	12 guides	£38	£4
Printing - dance instructor guide	£15.90/guide	12 guides	£191	£21
Recurrent programme delivery resources				
Programme delivery^c^	£32/hour	439 hours	£14,040	£1,560 (£53)
Travel expenses for programme delivery^b^			£2,915	£324 (£130)
Printing materials for programme delivery^d^			£2,013	£224
Indicative total cost^e^			£21,613	£2,401 (£104)
Indicative total costs (excluding one-off training)			£19,197	£2,133 (£139)
Total cost per girl (95 % CI)^f^			£73 (£71-£75)	

^a^Excluded from indicative total cost.

^b^Dance instructors could claim up to £10 travel expenses per session, average travel expenses claimed for induction training, booster training and programme delivery = £333 (SD = £136).

^c^Dance instructors were paid £32.00 per hour (each dance session was 1.25 hours in duration), sessions claimed ranged from 2–71 sessions, average sessions claimed 34 (SD = 19). Additional programme delivery expenses were claimed by dance instructors if they had provided cover for another dance instructor during programme delivery.

^d^Registers, dance diaries, spring half term reminder cards, Easter reminder cards, summer half term reminder cards, post-intervention dance booklets.

^e^Mainstream implementation of the programme would not include recruitment and marketing costs and were therefore excluded from the indicative total cost of BGDP.

^f^Average cost per school / maximum number of girls recruited from each school (*n* = 33).

## Discussion

In this school-based cluster randomised controlled trial we found no evidence that an after-school dance programme had any effect on accelerometer-assessed physical activity in the after-school period or overall physical activity of Year 7 girls, either while the programme was running or 12 months after the baseline assessment. We also found that only a third of the girls allocated to the intervention group met the pre-set adherence criteria of attending two thirds of the sessions provided in their school. We showed that when the data were re-analysed using a CACE per-protocol analysis there was no evidence of difference in weekday MVPA while either the programme was running or 12 months after baseline data were collected and the programme had ceased. These findings were largely unaltered in further sensitivity analyses. We also showed that BGDP is of comparable cost to other school-based PA interventions [53]. Exploratory analysis showed that girls allocated to the intervention arm who attended dance classes on the days of measurement obtained 4.7 more minutes of MVPA, 14.2 more minutes of light intensity physical activity and 258 more accelerometer counts per minute between 15.00 and 17.00 on dance days versus non-dance days. Data suggest that the impact on MVPA was relatively small and would have been diluted after accounting for non-dance days. There was evidence of a difference in all of the SDT-based motivation scores at T2, except autonomy need satisfaction and self-esteem, with higher scores in the control group.

Additional exploratory analyses showed that girls in the intervention arm who attended the dance sessions obtained 15 more minutes of light intensity physical activity and 4.7 more minutes of MVPA when compared to MVPA on days that the clubs did not run. This suggests that for the 1/3 of the girls adhering to the intervention the dance programme was a contributing source of physical activity. However, the level of activity was lower than anticipated, suggesting that session intensity needed to be greater in order to impact on MVPA at the T1 assessment. This finding implies that the dance classes provided physical activity but the session intensity at the point of measurement was lower than we anticipated. This low level of activity may reflect the stage of the intervention as many intervention groups were preparing for a dance performance at this time and it is conceivable that levels of activity during performance preparation may be lower (e.g., rehearsing, watching, and discussing) than during general sessions when activity dominates. A recent US study has reported that adolescent girls obtained an average of 17 minutes of MVPA from a dance class and that there was scope for further increases in the MVPA obtained from the session [54]. As such, the study findings are consistent with previous studies, which have shown that it is possible to deliver after-school programmes, and that dance can provide physical activity but more work is needed to optimise the intensity of the sessions. Moreover, the amount of MVPA that was obtained by the girls in the intervention arm at T1 is likely to be an underestimate and may have been higher if the girls were not preparing for dance performances. Equally, it may also be the case that the accelerometers were unable to capture the twisting, turning and bending that were part of the dance classes and as such activity in the dance classes is underestimated. Collectively, the data suggest that for some girls dance is a viable form of physical activity but the potential public health utility of this intervention approach could not be elucidated as we do not know if it is limited because of the attendance levels. This finding suggests that schools should consider offering dance programmes dance sessions which are not as high an attendance commitment than BGDP (i.e., perhaps fewer weeks and once per week.

A number of previous studies have shown that it is possible to deliver effective physical activity interventions in the after-school period [15, 18, 55–57]. The majority of these studies have originated from the US and have focussed on increasing capacity in pre-existing programmes by training the staff, who are either school staff or coaches from well-established programmes such as the YMCA, to increase the quality of the physical activity provided. This option was not possible in the UK school system where after-school provision is inconsistent varying in terms of the number of clubs offered, duration and quality both within and between schools. UK after-school provision often consists of “clubs” that are focussed on competitive invasion games such as football, rugby, netball or hockey and generally do not include dance [58]. In this context, the results of this study show that it is possible to instigate new after-school clubs in the UK, but the content of the sessions needs to be optimised to maintain attendance, session intensity and effect on habitual physical activity.

The lack of a difference in weekday MVPA between the intervention and control groups at T1 and T2 could have been a function of the characteristics of the girls who were recruited into the study who were reasonably active at baseline; the intervention and control group girls obtained 53 and 49 minutes of weekday MVPA respectively. As noted above, it has been suggested that the greatest reductions in all-cause mortality and other health benefits will be achieved by helping individuals who are currently inactive to achieve moderate increases in MVPA [4, 5]. However, baseline physical activity levels suggest that girls were already reasonably active and therefore the potential to increase MVPA was limited. Thus, these findings might suggest that there is a need to target physical activity interventions at children with lower levels of physical activity. It also leads to the conclusion that it may be beneficial for clinicians to work with physical activity professionals in order to identify the children in greatest need of increases in physical activity.

The girls recruited to the study were considerably more active than the girls who took part in the feasibility study, who obtained an average of 35 minutes of MVPA per day at baseline [20]. Identifying the reasons for this considerable difference is difficult. In the current study the girls received a taster session as part of the recruitment campaign. This session was delivered by independent dance instructors, was standardised across schools, and focussed on showing that dance could be an enjoyable activity in which all girls, regardless of previous dance experience, could enjoy. It was therefore specifically intended to encourage girls who were less physically active to join the study and staff perceptions are that we recruited a cross-section of girls with a range of different levels of physical activity. The only other difference in terms of recruitment between the feasibility study and the current study was the timing of the recruitment. In the current study, recruitment was conducted at the start of the autumn term when the girls had only recently joined the school. In the feasibility study, recruitment was conducted at the start of the spring term of Year 7 and it is possible that this delay allowed time for the more active girls to join other team sports programmes within the school.

Analysis of secondary psychosocial variables showed that both autonomous and controlled motivation for physical activity and dance was lower in the intervention group than the control group at T1 and T2. The reduction in controlled motivation suggests that intervention girls felt less motivated by guilt or external contingencies, which is motivationally adaptive and partially consistent with our hypotheses. However, the concurrent reduction in autonomous motivation in the intervention group is suggestive of an overall reduction of motivational quantity, which is not what we hypothesised. Perceptions of competence and relatedness were also lower in intervention versus control group girls, which was not expected within the context of a need–supportive intervention. Potential explanations include low intervention theory fidelity (which will be investigated in the process evaluation) and the timing of the measures (i.e., when girls were preparing for a performance which may have threatened perceptions of competence or social disagreements between girls).

### Relation to other studies

A 2012 meta-analysis of physical activity interventions that used an objective assessment of physical activity reported strong evidence of a small effect on MVPA of approximately four additional minutes per day [13]. The authors of that review suggested that the relatively small effect of physical activity interventions could be because girls in the intervention group swap one form of activity for an equally intense form of physical activity. However, examination of participant after-school activities between 15:00 and bedtime for intervention and control group girls provided no descriptive evidence of a difference between groups across all types of activities. There was, however, limited evidence of a small difference in dance participation between the two study arms at T1 (36 % control, 31 % intervention) which was inverted at T2 (30 % control, 34 % intervention). This might suggest that a small proportion of girls in the intervention arm did not participate in additional dance activities during the intervention period but when the dance sessions had stopped they took part in more dance. This finding may suggest that any trading of behaviours due to attending extra-curricular programs is limited to the focus of the extra-curricular club and approaches that focus on more general, non-specific forms of physical activity may hold greater potential.

A number of studies have suggested that dance holds promise as a means of engaging girls in physical activity and small studies have proposed a number of physical and mental benefits of dance [24–26, 59, 60]. The potential of this study to provide information on the long-term physical and mental health benefits of dance for adolescent girls is limited because only 1/3 of the intervention girls met the pre-specified attendance criteria. As such it is not possible to use the data from this project to assess the effect of attending dance programmes on self-esteem or other health outcomes. The qualitative elements of this project, which will be reported elsewhere, showed that children enjoyed the sessions and valued the content of the programme. Attendance was influenced by the days that the programme ran, the duration of the programme, school support for the programme and competing activities at the school and these are all issues that could be addressed in future after-school programmes.

### Strengths and weaknesses of the study

This study was carefully designed to address limitations of previous evaluations of school-based after-school physical activity interventions. Specifically, the protocol was published before the study started, schools were the unit of assignment, schools were randomised after baseline data were collected, and objective assessments of physical activity were obtained during the intervention period and 12 months after the baseline data had been collected. The intervention was developed over a five year period in accordance with the MRC framework for the evaluation of complex behavioural interventions [61] and intervention components are reported in accordance with the TIDieR guidelines [37]. Our sample size calculations indicated that 432 girls would need to be retained in the final sample to provide 90 % power (5 % alpha) to detect a ten-minute difference in weekday MVPA with a school-associated intra-class correlation of 0.087. The final sample for the T2 analysis (primary outcome) included 508 girls, and the ICC for the T2 intention to treat analysis was <0.001. There was ample power to detect a change, however there was no evidence of a difference between trial arms. We also provided detailed information on the costs of intervention delivery, enabling other researchers and school staff to compare the costs of this after-school programme with other options. It is important to note that we intended to conduct cost-effectiveness analysis but as the intervention was not effective, such analysis is not meaningful in understanding the findings of this study and has therefore not been presented. The study design could have been improved by collecting additional information such as MVPA during the middle of the programme and during a dance session. It would also have been informative to assess whether school travel mode changed as a result of attending the dance programme because parents were able to collect from school at 4.45 as opposed to the usual 3.30 pm which may have further attenuated any differences in weekday MVPA at T1.

## Conclusions

This trial showed no evidence that an after-school dance programme can increase the physical activity of Year 7 girls. The findings from this study raise a number of unanswered questions for physicians, public health practitioners and researchers. The most important question is how can we help adolescent girls to be physically active? We developed this study because we hypothesized that dance, an activity that many girls have said they enjoy, would be a useful means of encouraging greater levels of physical activity. However, this dance-based intervention, in which only 1/3 of the girls attended 2/3 of the sessions, had no impact on physical activity during the programme or 12 months after the baseline data had been collected. The data presented in this paper also suggests that there was little evidence of physical activity compensation in which children swap one activity for another, but we did not assess whether school travel mode, a key source of physical activity for adolescent girls [62] changed as a result of attending the dance classes. A more in-depth examination of changes in physical activity patterns as a result of participating in defined activities may therefore be warranted. In light of the results of this study, a key challenge for future research is to find ways to establish sustainable after-school programmes and optimise the delivery within these settings. Such an approach would significantly enhance external validity but would be reliant on the establishment of consistent after-school provision in UK secondary schools. In addition, it is necessary to understand how to engage inactive girls in physical activity interventions. More work is needed to find ways to help adolescent girls to be physically active. Crucially we need to either deliver activities such as dance in a way that girls can adhere to and/or identify alternative activities that adolescent girls will maintain.

## Additional file

Additional file 1:
**Supplementary Table A.** The Bristol Girls Dance Project intervention details - TIDieR checklist. **Supplementary Table B.** Data provision for participants at each time point by trial arm. **Supplementary Table C.** Means (SD) for physical activity variables intervention girls attending and not attending dance classes during measurement period between 3 and 5 pm. **Supplementary Table D.** After-school activity engagement of girls at T0. **Supplementary Table E.** Engagement in dance activities at T1 and T2. **Supplementary Table F.** Afterschool (3.30pm-bedtime) activity engagement during the last week. **Supplementary Table G.** Health outcomes measured in BGDP - responses to the EQ-5D-Y descriptive system. (DOCX 47 kb)
