# Supplementary material for: Effect and cost of an after-school dance programme on the physical activity of 11–12 year old girls: The Bristol Girls Dance Project, a school-based cluster randomised controlled trial
Source: Int J Behav Nutr Phys Act. 2015 Oct 6;12:128. doi: 10.1186/s12966-015-0289-y (PMC4595057; doi:10.1186/s12966-015-0289-y)
Supplement: Additional file 1: — Supplementary Table A. The Bristol Girls Dance Project intervention details - TIDieR checklist. Supplementary Table B. Data provision for participants at each time point by trial arm. Supplementary Table C. Means (SD) for physical activity variables intervention girls attending and not attending dance classes during measurement period between 3 and 5 pm. Supplementary Table D. After-school activity engagement of girls at T0. Supplementary Table E. Engagement in dance activities at T1 and T2. Supplementary Table F. Afterschool (3.30pm-bedtime) activity engagement during the last week. Supplementary Table G. Health outcomes measured in BGDP - responses to the EQ-5D-Y descriptive system. (DOCX 47 kb) [file 12966_2015_289_MOESM1_ESM.docx]

**Supplementary Table A: The Bristol Girls Dance Project intervention details - TIDieR checklist**

| **Item** | **Description** |
| --- | --- |
| Name (1) | Bristol Girls Dance Project (BGDP) [locally known as ‘Active7’) |
| Why (2) | The primary aim was to assess the effectiveness and cost-effectiveness of an after-school dance intervention in increasing mean weekday minutes of moderate-to-vigorous physical activity (MVPA) among 11-12 year old (Year 7) girls. The proportion of adolescent girls who meet the recommended levels of MVPA per day is lower than for boys. Physical activity (PA) levels among children decline as they progress through to adolescence.  Girls lose interest in physical education, face barriers to being active and lack opportunities for other desired PA. Competence in PA declines throughout school and self-presentational concern can deter girls from PA. Dance is a desirable activity for girls and can contribute to MVPA. It provides opportunities for peer support and socialisation and is perceived as enjoyable.  The Active7 intervention is based on self-determination theory (SDT) which suggests that by satisfying individuals’ sense of freedom and choice, helping them feel competent in PA and supported by others, it will help nurture more self-determined forms of motivation to be physically active.  Research and current PA trends suggest there is a need for effective strategies to attenuate the decline in girls’ PA levels. A dance intervention has the potential to be a cost-effective way of engaging girls in PA. |
| What – Materials(3) | Training was provided for dance instructors who delivered the intervention. The content focused on the philosophy of the BGDP and how to deliver dance sessions underpinned by SDT. Specifically, the session covered different motivation and communication styles, how to create an autonomy-supportive environment, how to build a sense of belongingness among the group, and how to increase competence. A booster training session (mid-way through the intervention) included a refresher on motivating children and managing problem behaviour, as well as covering the remaining session plans.  All instructors were provided with a *Guide for Dance Instructors*, detailing the aims and objectives of the study and how to deliver sessions successfully. It also included 40 session plans. A gradual progression was incorporated into the intervention design.  All intervention school participants received a *dance diary*. This was a booklet for participants to reflect on their progression and the dance sessions (encouraging participants to reflect on what they had learnt and enjoyed, what they would like to do in future sessions and goals they wanted to achieve). |
| What - Procedures (4) | Secondary schools were recruited through a letter of invitation. A meeting was held between the school contact and BGDP Trial Manager to discuss logistical issues and obtain a signed study agreement form. A taster session was then delivered to all year 7 girls who were able to take part in PE. The taster sessions most often took place instead of PE lessons, and were led by one of the study’s trained dance instructors. Students were told about the study and given information packs for themselves and their parents. Children had to return signed consent forms a few days later in order to sign-up to the study.  There were 33 intervention places available per school. If more than 33 children provided consent, participants were randomly selected to participate. Reserves were used if a participant dropped out prior to baseline data collection.  Schools were randomly assigned to ‘intervention’ or ‘control’ groups and were made aware of their allocation shortly after baseline data collection (in order to allow sufficient time to organise the after-school sessions).  Intervention schools received dance sessions led by an external dance instructor. The dance sessions were free to the school and participants.  A process evaluation was conducted throughout the study to assess the implementation and mechanisms of intervention impact. The process evaluation reported on the dose, implementation and theory fidelity, and intervention receipt. Attendance was recorded by the instructor. Observations occurred at four occasions within a randomly chosen session within each quarter of the intervention to assess theory fidelity and intervention receipt. Participants recorded their perceived levels of enjoyment and exertion on four occasions |
| Who provided (5) | Freelance, female dance instructors from the local area were recruited to the study.  In total, ten instructors delivered the intervention. One instructor delivered the intervention in two schools. Half way through the intervention period one instructor withdrew and was replaced by a reserve. Instructors covered one another’s absences. Instructors were paid for each session they delivered. All instructors completed a half day training session, specific to the BGDP before the intervention began. Instructors also attended a half day ‘booster session’ after the first term of programme delivery. |
| How (6) | Dance sessions were open to a maximum of 33 participants, who consented to participate at the beginning of the study. |
| Where (7) | Dance session were delivered after-school in the nine intervention schools. Schools were located across the greater Bristol area. The sessions were delivered in school facilities, usually a dance studio or school hall. |
| When and how much (8) | Intervention schools received two 75 minute dance sessions per week (term-time only) for a period of 20 weeks (a total of 40 sessions), starting in January and finishing in June/July 2014. Sessions began 5-10 minutes after school (usually between 15.00 and 15.40). |
| Tailoring (9) | All instructors received the same training and materials to aid delivery. However, all were encouraged to adopt an autonomy-supportive environment in line with SDT. |
| Modifications (10) | No modifications were made to the intervention, however, after four months delivering the programme, one dance instructor was unable to continue. This instructor was replaced by a reserve instructor who delivered some taster sessions. |
| How well –  Planned (11) | Fidelity to the intervention manual was reported by dance instructors. Instructors indicated the extent to which the session was similar to the main features from manual as either ‘very’, ‘somewhat’ or ‘not at all’. |
| How well - Actual (12) | In total, 93 (26.80%) sessions were reported as being ‘very’ similar to the manual, 164 (47.26%) ‘somewhat’ similar and 90 (25.94%) ‘not at all’ (one session had missing data (0.29%). Instructor interviews revealed that adherence to the manual was initially high, but reduced over time in response to the needs and skills levels of the participants. No instructor fully adhered to the manual. Instructors reported adhering to the principles of SDT during the delivery of sessions by offering need-support; this aligned with participant views. |

**Supplementary Table B: Data provision for participants at each time point by trial arm**

|  | Control, n (%) | | Intervention, n (%) | Total n (%) |
| --- | --- | --- | --- | --- |
|  | T0 | | | |
| Height (cm) | 287 (50.26) | | 284 (49.74) | 571 (100.00) |
| Weight (kg) | 287 (50.26) | | 284 (49.74) | 571 (100.00) |
| Psychosocial questionnaire | 287 (50.26) | | 284 (49.74) | 571 (100.00) |
| EQ-5D-Y | 287 (50.26) | | 284 (49.74) | 571 (100.00) |
| Accelerometer returned | 286 (50.08) | | 282 (49.39) | 568 (99.47) |
| Valid weekday* | 276 (48.33) | | 273 (47.81) | 549 (96.15) |
| Valid weekend* | 221 (38.70) | | 210 (36.78) | 431 (75.48) |
|  | T1 | | | |
| Height (cm) | 284 (50.27) | | 281 (49.73) | 565 (100.00) |
| Weight (kg) | 284 (50.27) | | 281 (49.73) | 565 (100.00) |
| Psychosocial questionnaire | 284 (50.27) | | 281 (49.73) | 565 (100.00) |
| EQ-5D-Y | 284 (50.27) | | 281 (49.73) | 565 (100.00) |
| Accelerometer returned | 282 (49.91) | | 279 (49.38) | 561 (98.29) |
| Valid weekday* | 271 (47.96) | | 264 (46.73) | 535 (94.69) |
| Valid weekend* | 188 (33.27) | | 159 (28.14) | 347 (61.42) |
|  | T2 | | | |
| Height (cm) | 280 (50.09) | | 279 (49.91) | 559 (100.00) |
| Weight (kg) | 280 (50.18) | | 278 (49.82) | 558 (99.82) |
| Psychosocial questionnaire | 280 (50.09) | | 279 (49.91) | 559 (100.00) |
| EQ-5D-Y | 280 (50.09) | | 279 (49.91) | 559 (100.00) |
| Accelerometer returned | 280 (50.09) | | 277 (49.55) | 557 (99.64) |
| Valid weekday* | 270 (48.30) | | 254 (45.44) | 524 (93.74) |
| Valid weekend* | 170 (30.41) | | 150 (26.83) | 320 (57.25) |
|  |  | T0 + T1 | | |
| Accelerometer returned | 281 (49.21) | | 277 (48.51) | 558 (97.72) |
| Valid weekday** | 265 (46.41) | | 256 (44.83) | 521 (91.24) |
| Valid weekend** | 159 (27.85) | | 130 (22.77) | 289 (50.61) |
|  | T0 + T2 | | | |
| Accelerometer returned | 279 (48.86) | | 275 (48.16) | 554 (97.02) |
| Valid weekday** | 262 (45.88) | | 246 (43.08) | 508 (88.97) |
| Valid weekend** | 145 (25.39) | | 124 (21.72) | 269 (47.11) |

*Percentages are taken as a proportion of the total number of girls providing data at that time point.

**Percentages are taken as a proportion of the total number of girls providing data at T0.

**Supplementary Table C: Means (SD) for physical activity variables intervention girls attending and not attending dance classes during measurement period between 3 and 5 pm**

|  | Control (n = 280) | Intervention group attendees (n = 78) | | | | Intervention group non-attendees (n = 137) | | | |
| --- | --- | --- | --- | --- | --- | --- | --- | --- | --- |
|  | Mean weekday | Dance Day | Non-Dance Day | t^*^ | p | Dance Day | Non-Dance Day | t^*^ | p |
| Sedentary (min) | 63.18 (14.07) | 49.29 (14.01) | 63.92 (21.91) | -5.92 | <0.001 | 59.92 (19.37) | 60.94 (20.35) | -0.48 | 0.627 |
| Light (min) | 32.67 (8.94) | 45.94 (9.87) | 31.67 (12.77) | 9.40 | <0.001 | 33.69 (11.00) | 33.76 (13.59) | -0.60 | 0.952 |
| MVPA (min) | 15.78 (7.37) | 21.45 (8.44) | 16.84 (11.03) | 5.14 | <0.001 | 15.80 (10.85) | 14.00 (10.65) | 1.71 | 0.088 |
| CPM | 825.19 (435.57) | 1116.04 (360.78) | 858.06 (547.25) | 4.62 | <0.001 | 844.15 (503.33) | 784.85 (467.51) | 1.20 | 0.231 |

* Paired sample t-test for within subject differences on days participants should have been attending dance and non-dance weekdays

**Supplementary Table D: After-school activity engagement of girls at T0**

|  | Control | | Intervention | |
| --- | --- | --- | --- | --- |
|  | N (taking part in activity) | % (of 287) | N (taking part in activity) | % (of 284) |
| Sports Club | 110 | 38.33 | 125 | 44.01 |
| Activity | 52 | 18.12 | 70 | 24.65 |
| Playing on Own | 21 | 7.32 | 13 | 4.58 |
| Sitting Down | 34 | 11.85 | 21 | 7.39 |
| TOTAL | 217 | 75.61 | 229 | 80.63 |

**Supplementary Table E: Engagement in dance activities at T1 and T2**

|  | T1 | | | | T2 | | | |
| --- | --- | --- | --- | --- | --- | --- | --- | --- |
|  | Control | | Intervention | | Control | | Intervention | |
|  | N | % | N | % | N | % | N | % |
| No | 181 | 63.73 | 195 | 69.53 | 197 | 70.36 | 185 | 66.31 |
| Yes | 103 | 36.26 | 86 | 30.47 | 83 | 29.64 | 94 | 33.69 |
| Quantity (days) | | | | |  | | | |
| 1 | 37 | 35.92 | 23 | 26.74 | 20 | 24.10 | 34 | 36.17 |
| 2 | 28 | 27.18 | 23 | 26.74 | 29 | 34.94 | 21 | 22.34 |
| 3 | 15 | 14.56 | 17 | 19.77 | 10 | 12.05 | 14 | 14.89 |
| 4 | 9 | 8.74 | 8 | 9.30 | 7 | 8.43 | 8 | 8.51 |
| 5+ | 14 | 13.59 | 14 | 16.28 | 17 | 20.48 | 16 | 17.02 |

**Supplementary Table F: Afterschool (3.30pm-bedtime) activity engagement during the last week**

|  |  | T1 | | | | T2 | | | |
| --- | --- | --- | --- | --- | --- | --- | --- | --- | --- |
|  |  | Control | | Intervention | | Control | | Intervention | |
| Sports clubs | Weekday | N | % | N | % | N | % | N | % |
|  | 0 (days) | 71 | 25.00 | 71 | 25.27 | 66 | 23.57 | 82 | 29.39 |
|  | 1 | 55 | 19.37 | 42 | 14.95 | 66 | 23.57 | 59 | 21.15 |
|  | 2 | 62 | 21.83 | 64 | 22.78 | 57 | 20.36 | 47 | 16.85 |
|  | 3 | 41 | 14.44 | 35 | 12.46 | 39 | 13.93 | 36 | 12.90 |
|  | 4 | 19 | 6.69 | 20 | 7.12 | 21 | 7.50 | 18 | 6.45 |
|  | 5 | 36 | 12.68 | 49 | 17.44 | 31 | 11.07 | 37 | 13.26 |
|  | Weekend | | | | |  | | | |
|  | 0 (days) | 114 | 40.14 | 108 | 38.43 | 108 | 38.57 | 117 | 41.94 |
|  | 1 | 114 | 40.14 | 100 | 35.59 | 115 | 41.07 | 105 | 37.63 |
|  | 2 | 53 | 18.66 | 72 | 25.62 | 57 | 20.36 | 57 | 20.43 |
|  | Missing | 3 | 1.06 | 1 | 0.36 | - | - | - | - |
| Activity clubs | Weekday | | | | |  | | | |
|  | 0 (days) | 166 | 58.45 | 136 | 48.40 | 148 | 52.86 | 150 | 53.76 |
|  | 1 | 57 | 20.07 | 62 | 22.06 | 58 | 20.71 | 57 | 20.43 |
|  | 2 | 19 | 6.69 | 33 | 11.74 | 30 | 10.71 | 40 | 14.34 |
|  | 3 | 12 | 4.23 | 16 | 5.69 | 17 | 6.07 | 12 | 4.30 |
|  | 4 | 7 | 2.46 | 8 | 2.85 | 7 | 2.50 | 5 | 1.79 |
|  | 5 | 23 | 8.10 | 26 | 9.25 | 20 | 7.14 | 15 | 5.38 |
|  | Weekend | | | | |  | | | |
|  | 0 (days) | 204 | 71.83 | 178 | 63.35 | 181 | 64.64 | 192 | 68.82 |
|  | 1 | 42 | 14.79 | 64 | 22.78 | 69 | 24.64 | 59 | 21.15 |
|  | 2 | 34 | 11.97 | 35 | 12.46 | 30 | 10.71 | 28 | 10.04 |
|  | Missing | 4 | 1.41 | 4 | 1.42 | - | - | - | - |
| Playing on own or with friends | Weekday | | | | |  | | | |
|  | 0 (days) | 11 | 3.87 | 22 | 7.83 | 22 | 7.86 | 20 | 7.17 |
|  | 1 | 24 | 8.45 | 28 | 9.96 | 39 | 13.93 | 36 | 12.90 |
|  | 2 | 50 | 17.61 | 53 | 18.86 | 55 | 19.64 | 53 | 19.00 |
|  | 3 | 52 | 18.31 | 46 | 16.37 | 56 | 20.00 | 53 | 19.00 |
|  | 4 | 55 | 19.37 | 35 | 12.46 | 45 | 16.07 | 49 | 17.56 |
|  | 5 | 92 | 32.39 | 97 | 34.52 | 63 | 22.50 | 68 | 24.37 |
|  | Weekend | | | | |  | | | |
|  | 0 (days) | 21 | 7.39 | 26 | 9.25 | 31 | 11.07 | 27 | 9.68 |
|  | 1 | 86 | 30.28 | 82 | 29.18 | 109 | 38.93 | 105 | 37.63 |
|  | 2 | 175 | 61.62 | 171 | 60.85 | 140 | 50.00 | 146 | 52.33 |
|  | Missing | 2 | 0.70 | 2 | 0.71 | - | - | 1 | 0.36 |
| Sitting down activities | Weekday | | | | |  | | | |
|  | 0 (days) | 9 | 3.17 | 20 | 7.12 | 10 | 3.57 | 21 | 7.53 |
|  | 1 | 57 | 20.07 | 61 | 21.71 | 44 | 15.71 | 49 | 17.56 |
|  | 2 | 68 | 23.94 | 72 | 25.62 | 71 | 25.36 | 57 | 20.43 |
|  | 3 | 55 | 19.37 | 46 | 16.37 | 48 | 17.14 | 53 | 19.00 |
|  | 4 | 29 | 10.21 | 21 | 7.47 | 37 | 13.21 | 33 | 11.83 |
|  | 5 | 66 | 23.24 | 61 | 21.71 | 70 | 25.00 | 66 | 23.66 |
|  | Weekend | | | | |  | | | |
|  | 0 | 32 | 11.27 | 42 | 14.95 | 26 | 9.29 | 35 | 12.54 |
|  | 1 | 152 | 53.52 | 138 | 49.11 | 134 | 47.86 | 125 | 44.80 |
|  | 2 | 99 | 34.86 | 99 | 35.23 | 119 | 42.50 | 119 | 42.65 |
|  | Missing | 1 | 0.35 | 2 | 0.71 | 1 | 0.36 | - | - |

**Supplementary Table G: Health outcomes measured in BGDP - responses to the EQ-5D-Y descriptive system***

|  | *n* | Mean | *SD* | Median | 25% IQR | 75% IQR | P value** |
| --- | --- | --- | --- | --- | --- | --- | --- |
| **T0** | | | | | | | |
| Intervention | 278 | 0.92 | 0.12 | 1.00 | 0.80 | 1.00 | 0.309 |
| Control | 279 | 0.91 | 0.16 | 1.00 | 0.80 | 1.00 |  |
| Total | 557 | 0.91 | 0.14 | 1.00 | 0.80 | 1.00 |  |
| **T1** | | | | | | | |
| Intervention | 278 | 0.89 | 0.17 | 1.00 | 0.80 | 1.00 | 0.667 |
| Control | 279 | 0.89 | 0.18 | 1.00 | 0.80 | 1.00 |  |
| Total | 557 | 0.89 | 0.17 | 1.00 | 0.80 | 1.00 |  |
| **T2** | | | | | | | |
| Intervention | 278 | 0.91 | 0.17 | 1.00 | 0.80 | 1.00 | 0.382 |
| Control | 279 | 0.91 | 0.14 | 1.00 | 0.80 | 1.00 |  |
| Total | 557 | 0.91 | 0.15 | 1.00 | 0.80 | 1.00 |  |

*EQ-5D-Y scores range from 0.00 (dead) to 1.00 (perfect health). Notably, some health states are regarded as being worse than death and have negative valuations.

**Rank sum between groups.
